# Bone induction and defect repair by true bone ceramics incorporated with rhBMP-2 and Sr

**DOI:** 10.1007/s10856-021-06587-7

**Published:** 2021-08-24

**Authors:** Chunli Zhang, Gang Xu, Liwei Han, Xiantong Hu, Yantao Zhao, Zhonghai Li

**Affiliations:** 1 Department of Orthopedics, Fourth Medical Center of the General Hospital of PLA, 100048 Beijing, China; 2 Beijing Engineering Research Center of Orthopedics Implants, 100048 Beijing, China; 3 Department of Orthopaedics, First Affiliated Hospital of Dalian Medical University, 116011 Dalian, China; 4 Key Laboratory of Molecular Mechanism for Repair and Remodeling of Orthopaedic Diseases, Dalian, 116011 Liaoning Province China

## Abstract

Objective: To study the bone induction and defect repair of true bone ceramics (TBC) combined with rhBMP-2 and Sr. Methods: MC3T3-E1 cells were used to evaluate the bioactivity of the composite. Cell proliferation activity was detected by CCK-8, ALP activity was detected by p-nitrophenyl phosphate (PNPP), and the differences of material surface topography were observed by scanning electron microscopy (SEM). Bone induction was verified by the implantation in nude mice. The rabbit femoral condyle defect model was achieved to verify the bone defect repair ability of the material. Results: SEM results showed nearly the same surface morphology and cell proliferation quantified by CCK-8 showed that compared with TBC, both TBC&Sr and TBC&BMP-2&Sr had a significant promoting effect (*P* < 0.05). ALP activity result showed that the ALP activity of TBC&BMP-2&Sr was significantly higher than that of TBC alone (*P* < 0.05). The bone induction result showed that TBC&Sr had a small amount of new bone formation, and the new bone area was only 2.5 ± 0.11%. The bone induction activity of TBC&BMP-2&Sr was the highest, the new bone area was up to 75.36 ± 4.21%. Histological result of bone defect repair showed that TBC&BMP-2&Sr was also the highest, the new bone area was up to 72.42 ± 3.14%. The repair effect of TBC& BMP-2 was second, and better than that of TBC&Sr. Conclusion: TBC combined with rhBMP-2 and Sr had the good bioactivity, obvious bone conduction and bone defect repair performance, laying the foundation of clinical application potentially.

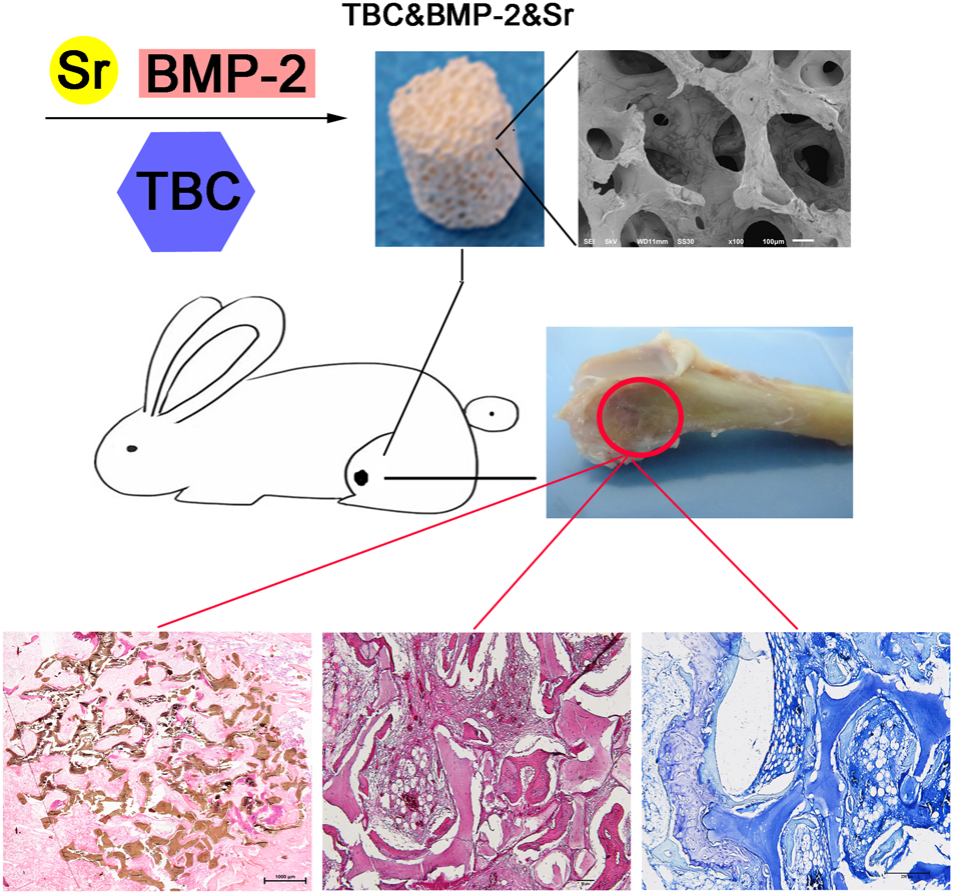

## Introduction

The clinical application of bone replacement materials is very promising [1], the research and development of which has always been the focus of the international orthopedic material field [2]. The xenograft bones such as true bone ceramics (TBC) possess the bioactivity and the pore structure for cells and tissues growing, which are good bone scaffold materials [3]. In addition, TBC has good bone conduction. However, as the autoimmune was removed as well as other osteogenic activity factors, TBC is usually with poor bone induction. Therefore, some active factors are added into TBC to boost the osteogenic activity.

In the study of osteogenic active elements, Strontium (Sr) can promote bone formation and inhibit bone absorption, the Sr compounds such as Sr Ranelate are currently effective drugs for the treatment of osteoporosis. Sr was incorporated into bone tissue engineering materials to improve the osteogenic activity of the original scaffold or filler material, such as Sr-doped tricalcium phosphate, hydroxyapatite, freeze-dried bone, and so on [4–6]. Sr-doped freeze-dried bone was previously prepared by our group via the means of aqueous solution and mild ion exchange, the highest proportion of Sr could reach about 10% [6]. The sustained release experiments showed that Sr could be released slowly for a long time in the solution [6].

Bone Morphogenetic Protein (BMP) is one of the signaling molecules that promotes the bone formation and induces the osteoblast differentiation. At present, rhBMP-2 obtained by genetic engineering technology has been approved by FDA, and is widely used in the treatment of spinal fusion and nonunion. However, the high cost of BMP-2 limits its application. In addition, the high concentration of BMP-2 is likely to cause side effects such as ectopic osteogenesis. Besides, the high concentration of BMP-2 leading to hollow bones during the bone repair has also been reported [7]. Therefore, it is suggested to reduce the dosage of BMP-2 as far as possible under the premise of ensuring the effect of bone repair to avoid local and systemic side effects in the clinical and cost down. In this study, the TBC combined with rhBMP-2 and Sr was constructed to enhance the effects of bone conduction and bone repair and make it with the excellent bioactivity for providing the advanced and safe material for the treatment of bone defect.

## Materials and methods

### Preparation of the scaffold

TBC was purchased from Beijing Ostersys Medical Technology Co., LTD. The rough preparation process was as follows, fresh bovine cancellous bone was taken as the raw material, and the soft tissue was removed off, and cut into 5 mm × 5 mm × 2 mm cancellous bone blocks and cancellous bone column with diameter of 7.5 mm and length of 10 mm, and washed by the high-pressure water gun and ultrasonic cleaner. Then it was deproteinized by 1% Trinton X-100 and 3% H_2_O_2_, cleaned repeatedly and dried. The dried bone pieces were placed in a muffle furnace for calcination at high temperature for 6 h, cooled down to the room temperature, washed by purified water with the pH 7.0–7.5, and freeze-dried.

The sterilized TBC was immersed in the sterilized 30 mM SrCl_2_·6 H_2_O solution for 14 days oscillation, ultrasonically washed by deionized water four times to remove residual SrCl_2_, and freeze-dried to obtain TBC&Sr, the Sr proportion of which could be above 5%. Then TBC and TBC&Sr were respectively immersed in the sterilized rhBMP-2 (Beijing Ruiquan Biotechnology Co., LTD) solution, overnight at 4 °C, and freeze-dried to obtain TBC&BMP-2 and TBC&BMP-2&Sr. The TBC, TBC&Sr, TBC&BMP-2 and TBC&BMP-2&Sr were sterilized by Co60 (25 kGy).

### Bioactivity detection

MC3T3-E1 cells were used to evaluate the bioactivity of the above materials. MC3T3-E1 cells were cultured with α-MEM solution and 10% fetal calf serum. 2 ml suspended droplets of MC3T3-E1 cells with a concentration of 2 × 10^4^/ml were added to a 12-well cell culture plate with the material.

For the CCK-8 detection, the four materials were placed in medium containing 10%FBS and soaked for 24 h at room temperature. MC3T3-E1 cells were cultured with the material for 72 h. After the original medium was discarded, the cells were washed once with PBS, the α-MEM medium containing 10% CCK-8 was added and incubated at 37 °C for 30 min. The absorbance was measured at a wavelength of 450 nm.

For the scanning electron microscopy (SEM) observation, MC3T3-E1 cells were cultured with the material for 24 h. After the original medium was discarded, the cells were washed once with PBS, and fixed with 2.5% glutaraldehyde for 4 h, freeze-dried, sprayed with gold to observe the cellular adhesion on the material.

### Alkaline phosphatase (ALP) detection

MC3T3-E1 cells were cultured for 7 days subsequently to evaluate ALP activity. After the original medium was discarded, the cells were washed once with PBS and lysed in 0.2% Triton X-100 according to the manufacturer’s protocol (Beyotime). A total protein of supernatants was measured by a bicinchoninic acid assay kit (Pierce). The absorbance was measured using a microplate reader (Thermo lab systems, America) at a wavelength of 405 nm. Data were normalized to the total cell protein and were expressed as μM of p-nitrophenol/min/mg protein.

### Evaluation of bone induction activity in vivo

#### Experimental animals

18 BALB/C nude mice (18–22 g, male) were provided and raised by the Fourth Medical Center of the PLA General Hospital. The animal experiments were approved by the Animal Ethics Committee of the Fourth Medical Center of the PLA General Hospital. The environment was free of pathogens, the ambient temperature was 22 °C, 12 h light/12 h dark cycle was guaranteed every day, the indoor humidity was 50–55%, and adaptive feeding was carried out for 1 week before the experiment. NIH guidelines (or for non-U.S. residents similar national regulations) for the care and use of laboratory animals (NIH Publication #85-23 Rev. 1985) have been observed.

#### Surgical procedures and observations

The bone induction activity of BALB/C nude mice was observed by the intermuscular space implantation. Induction experiments were performed in the intermuscular space of the legs, TBC, TBC&Sr, TBC&BMP-2 and TBC&BMP-2&Sr were implanted respectively, six in each group. The mice were anesthetic by the injection with 0.3% pentobarbital intraperitoneally, the material was implanted in the left hind limb muscle bag of nude mice under aseptic conditions. The muscle bag, fascia layer and skin were sutured and feeding alone. Four weeks after surgery, the tissues were obtained and fixed with 4% paraformaldehyde. After decalcification, dehydration, embedding and sectioning and so on, paraffin sections were made and hematein eosin (HE) staining was performed. The new bone formation in the implanted material area was observed under the microscope, and the new bone area was quantified from three different fields selected randomly.

### Evaluation of rabbit bone defect repair

#### Experimental animals

24 New Zealand big-eared rabbits (Adult rabbits weighing about 3.0 kg, male or female) were provided and raised by the Fourth Medical Center of the PLA General Hospital. The animal experiments were approved by the Animal Ethics Committee of the Fourth Medical Center of the PLA General Hospital. The environment was free of pathogens, the ambient temperature was 22 °C, 12 h light/12 h dark cycle was guaranteed every day, the indoor humidity was 50–55%, and adaptive feeding was carried out for 1 week before the experiment. NIH guidelines (or for non-U.S. residents similar national regulations) for the care and use of laboratory animals (NIH Publication #85-23 Rev. 1985) have been observed.

#### Surgical procedures

Repair experiment of bilateral femoral condyle defect in rabbits was performed. The New Zealand big-eared rabbit was anesthetic and supine fixed on the operating table, with the routine preparation of skin, disinfection and laying sterile hole towel. Longitudinal incision on the lateral femoral condyle of the hind limb was selected and soft tissues were separated. The periosteum was removed as far as possible with a periosteum stripper to reveal the condyles of femur, a circular hole with a diameter of 5 mm, depth of 10 mm was drilled through the middle part of the femoral condyle to achieve the model. The composite material was mixed with normal saline and singly implanted into the bone defect of the experimental rabbits. The incisions were rinsed with 80 thousand units of gentamicin injection. Sampling and observation were performed 6 weeks after surgery.

#### Observations

For the general observation, swelling, redness and secretion in the wound were observed. After the animals were sacrificed, the inflammatory response in bone graft area was also observed.

For the histomorphological observation, some bone tissues were not decalcified, and sectioned with a hard tissue slicer for histomorphological observation by HE staining. Some bone tissues were decalcified, paraffin sections were selected, and stained with HE and toluidine blue (TB) for observation. The new bone formation in the implanted material area was observed under the microscope, and three different fields were randomly selected to conduct semi-quantitative analysis on the new bone formation.

### Statistical analysis

The measurement data were expressed as mean ± standard deviation. SPSS 16.0 statistical software was used for statistical analysis, the statistical difference was tested by one-way ANOVA, and *P* < 0.05 indicated statistical difference.

## Results

### Bioactivity

**Fig. 1:**
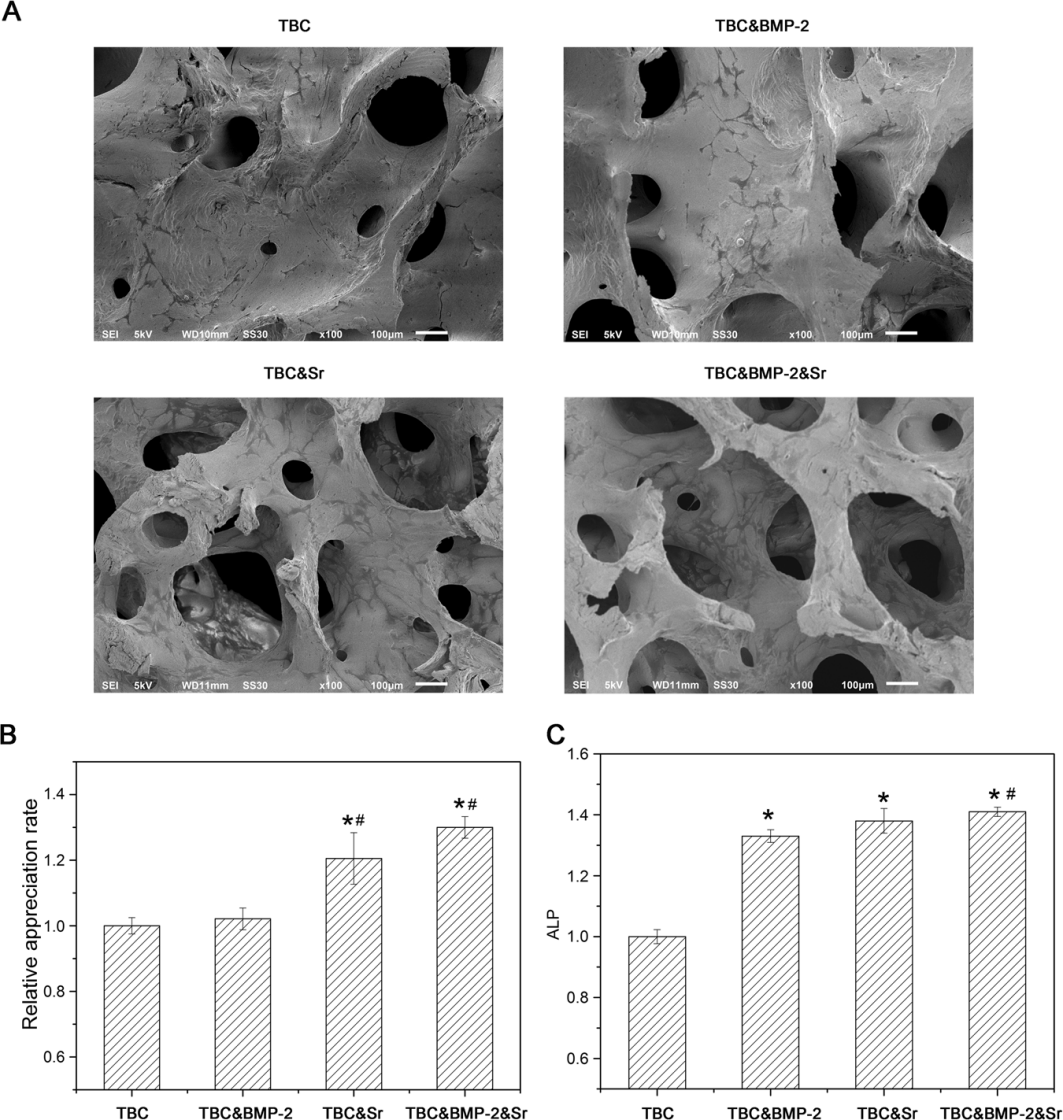
**A** The surface topography of the bone material observed by scanning electron microscopy (SEM) (100×); **B** Effects of four materials on the MC3T3-E1 proliferation (*n* = 4); **C** Effects of four materials on the ALP activity (*n* = 4); compared with TBC, *p < 0.05; compared with TBC&BMP-2, ^#^*p* < 0.05

The adhesion of cells on the surface of bone material may affect their bioactivity. SEM images of TBC, TBC&BMP-2, TBC&Sr, and TBC&BMP-2&Sr were examined to explore the difference of surface topography (Fig. 1A). It presented nearly the same surface morphology. Cell proliferation results detected by CCK-8 exhibited that both TBC&Sr and TBC&BMP-2&Sr obviously promoted the cell proliferation compared with TBC or TBC&BMP-2 (*P* < 0.05) (Fig. 1B), which were consistent with the SEM results, indicating the composite material has the good bioactivity. The ALP activity of cells 7 days after contact with the material showed that compared with TBC, ALP activities of TBC&BMP-2, TBC&Sr and TBC&BMP-2&Sr were all increased (*P* < 0.05) (Fig. 1C); compared with TBC&BMP-2 or TBC&Sr, that of TBC&BMP-2&Sr was also increased (*P* < 0.05) (Fig. 1C).

### Bone induction

**Fig. 2:**
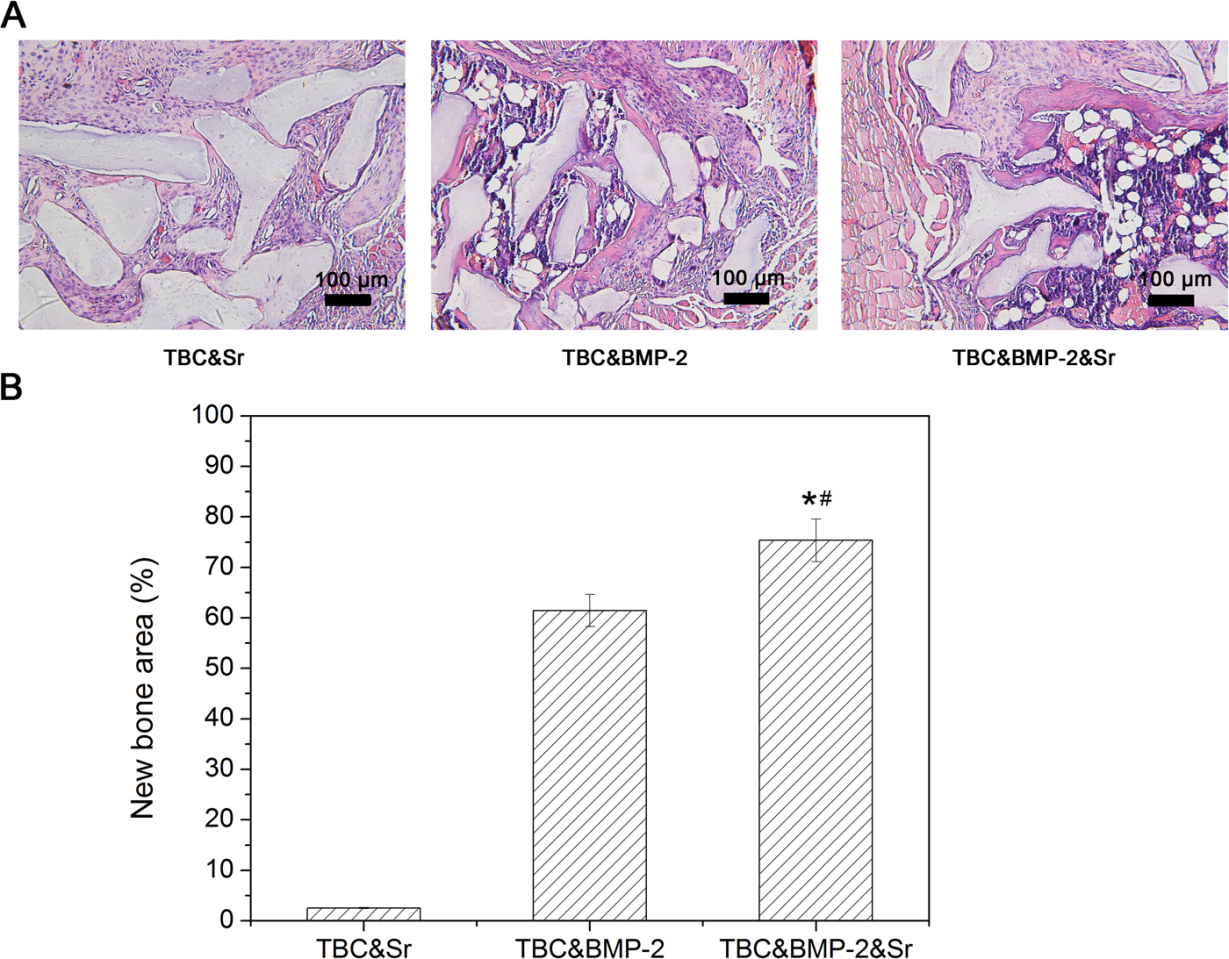
**A** Four weeks after the operation, the histology of each group was observed (HE staining, ×200); **B** Quantitative area of new bone formation (*n* = 3); compared with TBC&Sr, *p < 0.05; compared with TBC&BMP-2, ^#^*p* < 0.05

TBC has a good bone conduction effect, but no bone induction effect. After active factors were added, the bone induction activity was observed. The wounds of all animals were observed regularly after surgery. The results showed that there were no adverse reactions, such as swelling and exudation, and no obvious inflammatory reactions in the surgical site of mice in each group. The wounds healed well. The HE result showed that TBC&Sr had a small amount of new bone formation (Fig. 2A), the new bone area was 2.5 ± 0.11% (Fig. 2B); both TBC&BMP-2 and TBC&BMP-2&Sr presented obvious osteogenesis, like new bone, cartilage, and bone marrow - like cavity structures, the bone induction activity of TBC&BMP-2&Sr was better (Fig. 2A), the new bone area of which was up to 75.36 ± 4.21% (*P* < 0.05) (Fig. 2B).

### Bone defect repair

**Fig. 3:**
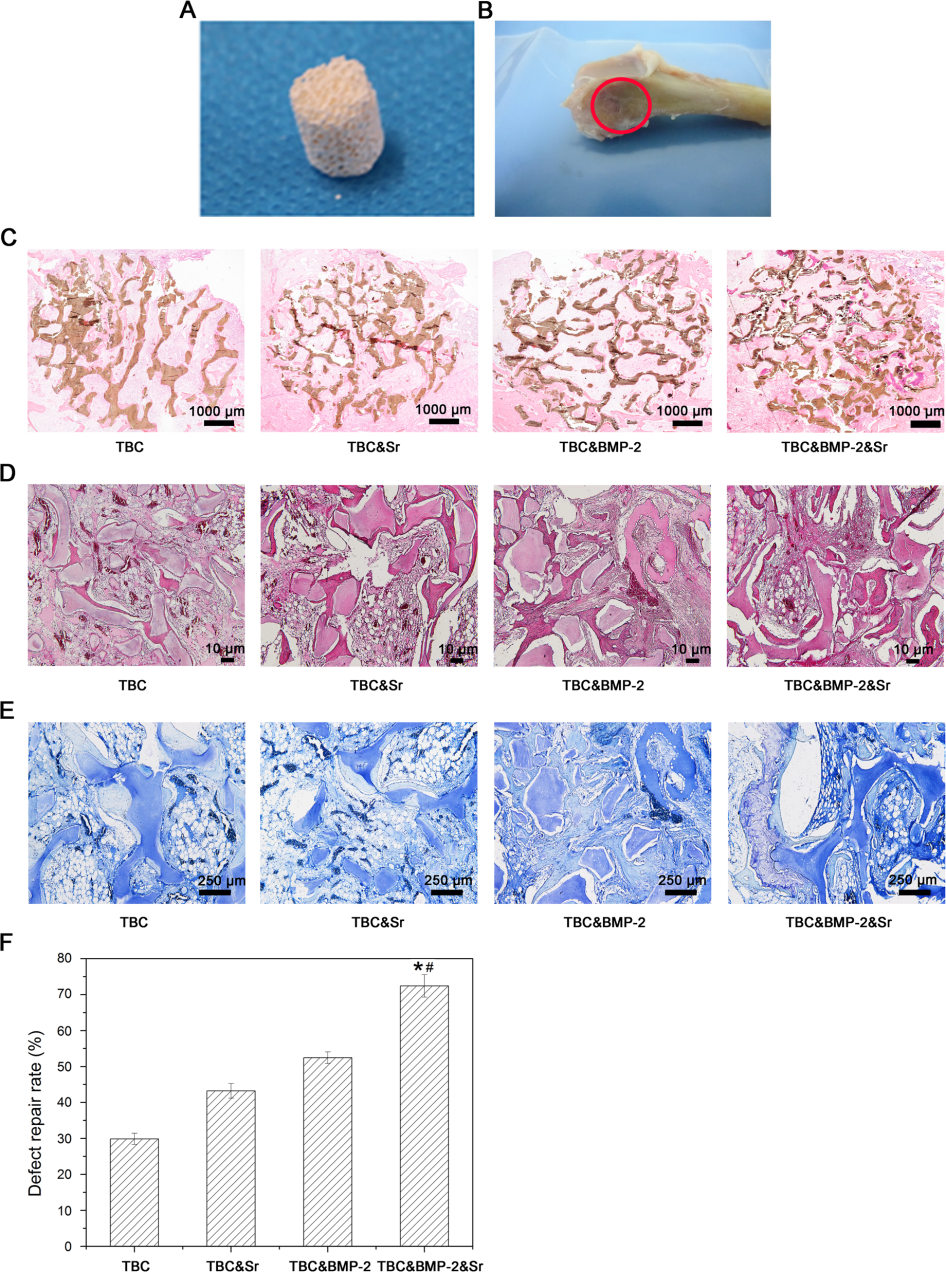
Rabbit femoral condyle bone repair experiment. **A** The appearance of experimental material; **B** The repaired bone 6 weeks after the surgery; **C** Hard tissue section (HE staining, 40×); **D** Paraffin section (HE staining, 100×); **E** Paraffin section (TB staining, 100×); **F** Quantitative results of the histological sections (*n* = 3); compared with TBC&Sr, *p < 0.05; compared with TBC&BMP-2, ^#^*p* < 0.05

The rabbit femoral condyle defect model was used to evaluate the repair ability of bone materials, the bone material was shown as Fig. 3A. All rabbit wounds were observed regularly after surgery, and the results showed no adverse reactions such as swelling and exudation, no obvious inflammatory reactions, good wound healing, and no death. After 6 weeks, the surgical site could be identified on the femur specimen (Fig. 3B). HE staining of hard tissue sections (Fig. 3C) showed that the scaffold wheel gallery of each group was brown and obviously residual, and the new bone regenerated around the scaffold, especially at the edge. HE staining of paraffin sections could also significantly reveal the observed TBC as well as the new bone matrix and fat cavity (Fig. 3D). And TB staining showed that the new bone was in dark blue (Fig. 3E). Further, based on the histological section results, the area of new bone was analyzed semi-quantitatively, indicating that the bone repair effect of TBC&BMP-2&Sr was the best, the new bone area could reach 72.42 ± 3.14% (*P* < 0.05) (Fig. 3F); the repair effect of TBC& BMP-2 was second, and both of which were better than that of TBC&Sr (*P* < 0.05) (Fig. 3F); TBC had the worst repair effect, the new bone area was 29.89 ± 1.54% (*P* < 0.05) (Fig. 3F).

## Discussion

The ideal bone graft should have good bioactivity and suitable biodegradability. In addition, it also has high requirements in bone conduction and bone inductivity [8]. Autologous bone is regarded as the “gold standard” in the study of many bone replacement materials, but it is often subject to the situation of less donor sources and postoperative discomforts [9]. Calcined heterogeneous bone made of bovine cancellous bone by calcination at high temperature is an ordered crystal of bone inorganic substance, the main component of which is high purity hydroxyapatite [10], and it has calcium - phosphorus ratio and pore structure similar to the human bone [11]. High temperature calcination not only effectively removes the antigen but also retains the natural porous structure, providing an ideal channel for the growth of new bone tissue and blood vessels. It is a potential ideal bone defect repair material.

In the field of bone regeneration, many metals or metal ions have been used in combination with bone replacement materials [12], such as calcium (Ca^2+^), copper (Cu^2+^), iron (Fe^2+^), magnesium (Mg^2+^), Sr (Sr^2+^), zinc (Zn^2+^) and so on. Sr plays a dual role in promoting bone formation and inhibiting bone resorption. Luo et al. [5] reported the Sr containing complex enhanced the osteogenic differentiation of mouse mesenchymal stromal cells in a dose-dependent manner by introducing various amounts of Sr into the amorphous apatite. Besides, it is also one of the most popular topics to combine metal ions with bioactive factors to improve the bone repair effect of the bone material. A low amount of fixed Sr (SrCO_3_ content < 10 wt%) contributed to rhBMP-2-inducing the osteogenic activity of Sr-doped calcium phosphate cement [13]. In C2C12 model cells, it was confirmed that the Sr-induced enhancement of bioactivity of rhBMP-2 was related to an elevated recognition of bone morphogenetic protein receptor-IA (BMPR-IA) to rhBMP-2 and an increased expression of BMPR-IA [13]. We also believed that the synergy effect of Sr and rhBMP-2 potentially helped to solve to the osteolysis caused by rhBMP-2 [14]. In this study, TBC&BMP-2&Sr improved the cells proliferation and ALP activity, indicating there was a certain synergistic effect between BMP-2 and Sr. In terms of cells adhere to the material, TBC&BMP-2&Sr also had more affinity for cells, which was consistent with the result detected by CCK-8. However, the effect of TBC&BMP-2&Sr on the expressions of osteoblast-related proteins and genes still remained to be further studied.

The BMP-2 protein has a powerful osteogenic effect [5, 14]. In this study, TBC obtained a certain bone induction ability by the addition of BMP-2, the new bone area was 61.44 ± 3.18%; the addition of Sr also improved the bone induction ability of TBC&BMP-2, and the new bone area increased from 61.44 ± 3.18% to 75.36 ± 4.21%, indicating the combined application of Sr and BMP-2 contributes to the bone induction of TBC. And the result was similarly to the report that the interaction between Sr and BMP-2 was found to accelerate the early osteogenesis in male C57BL/6 mice [15]. To the bone defect repair, the histology results showed that the scaffold wheel gallery of each group was obviously residual, the new bone regenerated around the scaffold, especially around the edges; TBC had the bone conduction ability, the repair effect of TBC alone was 29.89 ± 1.54%; the osteogenic capacity of TBC was increased by the addition of BMP-2 and Sr, the repair effect of TBC&BMP-2&Sr could reach up to 72.42 ± 3.14%, and was obviously higher than other groups (*P* < 0.05). These results suggested that both BMP-2 and Sr could improve the TBC repair ability, and BMP-2 was more effective; two factors could play a synergistic effect together, the specific action mechanisms were needed to be further studied.

## Conclusion

TBC combined with rhBMP-2 and Sr had the good bioactivity and osteogenic repair effect. BMP-2 and Sr could play a synergous role with obvious osteogenic effect and excellent bone defect performance, laying the foundation of clinical application potentially.
